# The Kidney Failure Risk Equation for Prediction of Allograft Loss in Kidney Transplant Recipients

**DOI:** 10.1016/j.xkme.2020.09.004

**Published:** 2020-10-28

**Authors:** Chi D. Chu, Elaine Ku, Mohammad Kazem Fallahzadeh, Charles E. McCulloch, Delphine S. Tuot

**Affiliations:** 1 Division of Nephrology, Department of Medicine, University of California San Francisco, San Francisco, CA; 2 Department of Epidemiology and Biostatistics, University of California San Francisco, San Francisco, CA

**Keywords:** Kidney transplant, risk prediction, kidney failure, clinical prediction models

## Abstract

**Rationale & Objective:**

The Kidney Failure Risk Equation (KFRE) is a simple widely validated prediction model using age, sex, estimated glomerular filtration rate, and urinary albumin-creatinine ratio to predict the risk for end-stage kidney disease. Data are limited for its applicability to kidney transplant recipients.

**Study Design:**

Validation study of the KFRE as a post hoc analysis of the Folic Acid for Vascular Outcomes Reduction in Transplantation (FAVORIT) Trial.

**Setting & Participants:**

Adult kidney transplant recipients with functioning kidney allografts at least 6 months posttransplantation from 30 centers in the United States, Canada, and Brazil. Participants with estimated glomerular filtration rates < 60 mL/min/1.73 m^2^ at study entry were included.

**Predictor:**

2- and 5-year kidney failure risk predicted by the KFRE using variables at study entry.

**Outcome:**

Graft loss, defined by initiation of dialysis.

**Analytical Approach:**

Discrimination of the KFRE was assessed using C statistics; calibration was assessed by plotting predicted risk against observed cumulative incidence of graft loss.

**Results:**

2,889 participants were included. Within 2 years, 98 participants developed graft loss, 107 participants died with a functioning graft, and 129 participants were lost to follow-up, and by 5 years, 252 had developed graft loss, 265 died with a functioning graft, and 1,543 were lost to follow-up. The KFRE demonstrated accurate calibration and discrimination (C statistic, 0.85 [95% CI, 0.81-0.88] at 2 years and 0.81 [95% CI, 0.78-0.84] at 5 years); performance was similar regardless of donor type (living vs deceased) and graft vintage, with the noted exception of poorer calibration for graft vintage less than 2 years.

**Limitations:**

Unavailable cause of graft loss.

**Conclusions:**

The KFRE accurately predicted the risk for graft loss among adult kidney transplant recipients with graft vintage longer than 2 years and may be a useful prognostic tool for nephrologists caring for kidney transplant recipients.

## Graphical abstract

**Figure:**
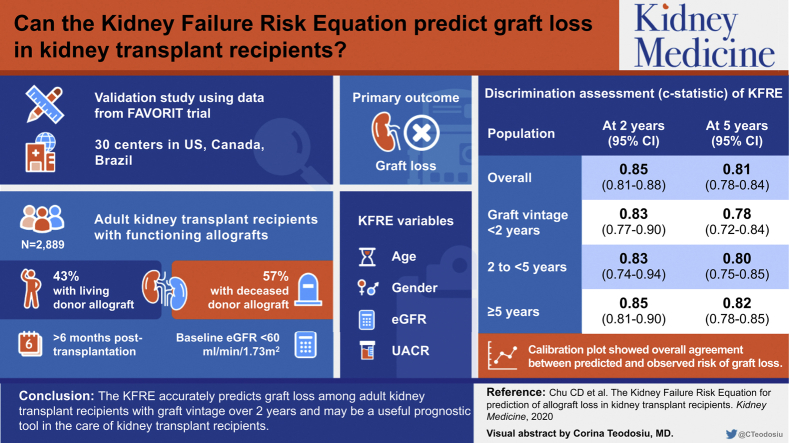

**Plain-Language Summary**

The Kidney Failure Risk Equation (KFRE) is a widely validated prognostic model using age, sex, estimated glomerular filtration rate, and urinary albumin-creatinine ratio to predict the risk for kidney failure within 2 and 5 years for patients with chronic kidney disease. In this study, we assessed the performance of the KFRE among 2,889 kidney transplant recipients for predicting the risk for kidney allograft failure with return to dialysis. We found that the KFRE accurately predicted the risk for kidney failure for kidney transplant recipients who were more than 2 years posttransplantation. The KFRE may be a useful prediction tool for clinicians caring for kidney transplant recipients years after transplantation when data for more complex prediction models are unavailable.

The Kidney Failure Risk Equation (KFRE) is a prediction model for calculating the risk for progression to kidney failure among adults with chronic kidney disease (CKD).^1^ The original risk equation was developed from 2 Canadian cohorts of patients with CKD referred to nephrology with estimated glomerular filtration rates (eGFRs) < 60 mL/min/1.73 m^2^, with prediction of dialysis initiation or preemptive kidney transplantation as the outcome. In external validation studies, the KFRE has demonstrated high accuracy in predicting progression of CKD to kidney failure in diverse multinational settings, primary care populations, and children with CKD.^2^, ^3^, ^4^, ^5^, ^6^ The 4-variable version of the KFRE uses age, sex, eGFR, and urinary albumin-creatinine ratio (UACR); an 8-variable version exists that adds serum bicarbonate, albumin, calcium, and phosphorus levels. These variables are routinely collected in CKD care, enabling integration of the KFRE into electronic health records and facilitating its application for risk-based clinical decision making, such as for triage of nephrology referrals and dialysis access planning.^7^, ^8^, ^9^, ^10^

Predicting allograft failure in the kidney transplant population has been a question of interest to inform clinical decisions such as planning for dialysis access and consideration for retransplantation. Although numerous prediction models have been previously developed for predicting CKD progression in kidney transplantation, none have been used widely to date.^11^, ^12^, ^13^, ^14^, ^15^ Most previously developed prognostic models for kidney transplant were developed and validated to make predictions at or shortly after the time of transplantation.^15^^,^^16^ By contrast, one model (iBox) has been validated to be predictive at any time after transplantation.^17^ Because kidney transplant recipients who have had stable kidney function years after transplantation may not have recent donor-specific antibody or histopathologic data (both inputs to iBox), the rationale of the study was to assess the performance of the KFRE as a model that may be useful and potentially already familiar to community nephrologists caring for kidney transplant recipients.

## Methods

We studied participants enrolled in the Folic Acid for Vascular Outcome Reduction in Transplantation (FAVORIT) Trial. Details of the study design have previously been described.^18^ In brief, the FAVORIT study was a multicenter randomized controlled trial designed to study the effect of folic acid, vitamin B_6_, and vitamin B_12_ supplementation on cardiovascular outcomes in kidney transplant recipients with elevated total homocysteine levels. Participants were randomly assigned to either a multivitamin containing a high-dose combination of folic acid, vitamin B_6_, and vitamin B_12_ or a multivitamin containing no folic acid and low doses of vitamin B_6_ and B_12_ based on estimated average requirement values. A total of 4,110 participants were enrolled from 30 clinical sites (27 in the United States, 2 in Canada, and 1 in Brazil) from 2002 through 2007, and follow-up ended in 2009.

The study protocol was approved by the institutional review board at each site, and written informed consent was obtained from all participants. Because the FAVORIT trial did not show statistically significant differences in either all-cause mortality or graft loss, we treated the trial as a cohort study for all analyses.^19^

### Study Population

FAVORIT recruited prevalent kidney transplant recipients aged 35 to 75 years who had a functional allograft for at least 6 months. To be eligible, participants had to have stable kidney function, defined initially as estimated creatinine clearance ≥ 30 mL/min but redefined in 2005 as ≥30 mL/min in men and ≥25 mL/min in women. An elevated serum homocysteine level was also required (≥12 μmol/L for men and ≥11 μmol/L for women) for inclusion.

For our study, we excluded participants with eGFRs ≥ 60 mL/min/1.73 m^2^ at the baseline study visit because the KFRE was derived and validated among persons with eGFRs below this level. We also excluded participants with missing baseline UACRs (n = 290) or missing eGFRs (n = 51), which are required for the KFRE. Although urinary protein excretion has sometimes been substituted in the KFRE for validation studies when urinary albumin excretion was unavailable, urinary protein testing was not performed in the study protocol and therefore could not be used when UACR was missing.^3^ Because urinary albumin and creatinine testing were performed during the baseline but not during follow-up study visits, participants could not be incorporated into the cohort if eGFR decreased to <60 mL/min/1.73 m^2^ at a later study visit.

### Variables

Demographic and clinical characteristics were collected for participants during their baseline study visit, which defined the start of follow-up time for each participant. The CKD Epidemiology Collaboration (CKD-EPI) equation was used to calculate eGFR.^20^ Laboratory methods for measuring serum creatinine, urinary creatinine, and urinary albumin have been previously described.^21^ Using the eGFR and UACR from the baseline visit, the 2- and 5-year risks for end-stage kidney disease for each participant were calculated using the 4-variable KFRE (Item S1). The North American KFRE equation was used for participants in the United States and Canada; the non–North American KFRE equation was used for those in Brazil. The KFRE-predicted risk for end-stage kidney disease served as the primary predictor.

### Outcomes

Participants were followed up every 6 months by alternating telephone interviews and clinic visits to obtain study outcomes, including ascertainment of death and dialysis initiation. Outcome ascertainment was supplemented by review of administrative and medical records as necessary. The primary outcome for this study was graft loss, defined by initiation of maintenance dialysis. Data for pre-emptive retransplantation were not available in the FAVORIT study data, and pre-emptive transplantation was not classified as graft loss according to the study protocol. Follow-up for outcome ascertainment ended on death, loss to follow-up, or administrative censoring at trial conclusion on June 24, 2009. The occurrence of a nonfatal cardiovascular event (ie, a primary outcome of FAVORIT) was not treated as a censoring event.

### Statistical Analysis

Predictive performance of the KFRE was assessed using metrics for discrimination and calibration. Following the usual recommendations for prediction model validation in survival analysis,^22^ we used the entire follow-up period to assess discrimination and calibration at 2 and 5 years. We assessed discrimination by computing C statistics with 95% CIs determined using a bootstrap approach with 500 repetitions.^23^ The 2- and 5-year C statistics represent the proportion of all pairs of participants, at least 1 of whom developed graft loss within 2 or 5 years, respectively, for which the KFRE assigned a higher risk to the participant who developed graft loss earlier. To account for the competing risk for death, we used an approach described by Wolbers et al^24^ for computing C statistics in the presence of competing events based on the Fine and Gray model.^25^ In this approach, participants are not censored at the occurrence of a competing event but instead are retained in the risk set beyond the end of the maximum observed follow-up time.

Calibration was assessed graphically by plotting observed risk for graft loss versus mean KFRE-predicted risk within previously proposed categories of predicted risk: 0% to <2%, 2% to <6%, 6% to <10%, 10% to <20%, and ≥20% for 2-year risk; and 0% to <3%, 3% to <5%, 5% to <15%, 15% to <25%, 25% to <50%, and ≥50% for 5-year risk.^2^ Observed risk for graft loss was obtained by estimating the cause-specific cumulative incidence of graft loss at 2 and 5 years, with death treated as a competing risk.^26^ Although treatment of death as a competing event did not result in significant differences in the initial derivation of the KFRE,^1^ we treated death as a competing event in our primary analysis because the cumulative incidence obtained in this manner has been proposed to have a more suitable interpretation for clinical risk prediction.^27^, ^28^, ^29^, ^30^

We assessed discrimination and calibration in the overall cohort and in prespecified subgroups based on donor type (living vs deceased) and graft vintage (categorized as <2, 2-<5, and ≥5 years since transplantation). Donor type may affect predictive performance given that living donor allografts have consistently shown longer graft survival compared with deceased donor allografts.^31^ We chose to examine subgroups based on vintage because differences in the risk and causes of graft failure in earlier versus later posttransplantation periods could affect predictive performance of the KFRE. Specifically, because early graft failure is often related to rejection, the KFRE, which does not include immunologic data, may not be as predictive.^32^ In addition, evidence for accurate prediction at longer graft vintage is important because most prediction models for graft failure have been validated in the early posttransplantation period and have not been validated in later posttransplantation periods, with the exception of the iBox model of Loupy et al.^17^

Study data were obtained from the National Institute of Diabetes and Digestive and Kidney Diseases Central Repository in deidentified form. The University of California, San Francisco Institutional Review Board considers this study exempt human subjects research. We followed guidelines for reporting validation of a risk prediction model as described by the Transparent Reporting of a Multivariable Prediction Model for Individual Prognosis or Diagnosis (TRIPOD) Statement (Item S2).^33^ All analyses were performed using R, version 3.6.1 (R Foundation for Statistical Computing), and Stata/IC, version 15.1 (StataCorp).

### Sensitivity Analyses

As sensitivity analysis, we assessed discrimination and calibration treating death as a censoring rather than a competing event, as in the original derivation of the KFRE, using Cox proportional hazards models. In an additional sensitivity analysis, we performed analyses in which we included the 880 participants with eGFRs ≥ 60 mL/min/1.73 m^2^, truncating these values to 60 for calculation of the KFRE.

## Results

**Figure 1:**
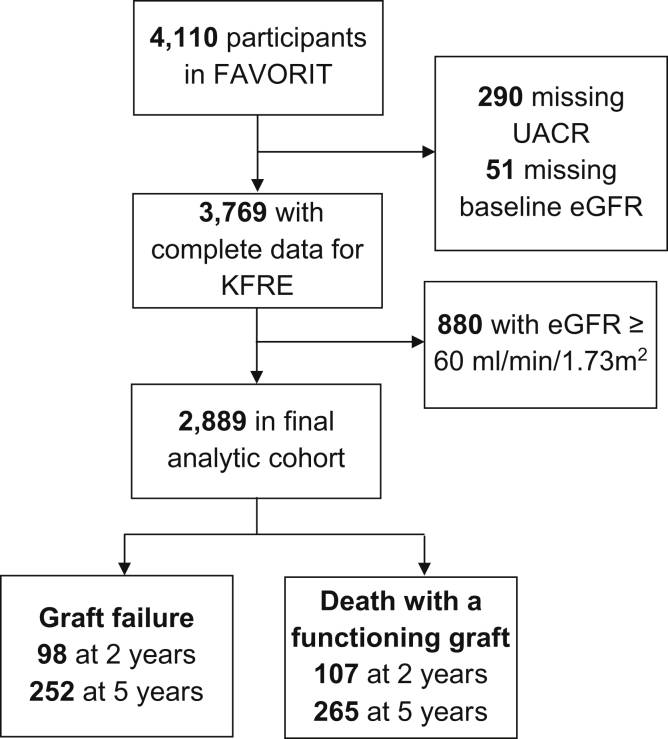
Study flow diagram. Abbreviations: eGFR, estimated glomerular filtration rate; FAVORIT, Folic Acid for Vascular Outcomes Reduction in Transplantation Trial; KFRE, Kidney Failure Risk Equation; UACR, urinary albumin-creatinine ratio.

Of the 4,110 participants in FAVORIT, a total of 1,221 were excluded due to missing baseline data or eGFR ≥ 60 mL/min/1.73 m^2^ (Fig 1). Baseline characteristics for 2,889 included participants are shown in Table 1. Mean age was 52.2 (standard deviation [SD], 9.3) years. Mean eGFR was 41 (SD, 11) mL/min/1.73 m^2^ and median UACR was 28 (interquartile range [IQR], 10-119) mg/g. A total of 43% of participants (n = 1,229) received kidney transplants from a living donor and 57% (n = 1,633) had deceased donor transplants. Median graft vintage was 4.3 (IQR, 1.7-8.0) years; the distribution is shown in Figure S1.

**Table 1:** Validation Cohort Baseline Characteristics

Characteristic	Parameter Estimate
Age, y	52.2 (9.3)
Female sex	1,127 (39.0%)
Race	
American Indian or Alaska Native	24 (0.8%)
Asian	59 (2.1%)
Black	456 (15.9%)
Mixed	100 (3.5%)
Native Hawaiian or Pacific Islander	4 (0.1%)
White	2,226 (77.6%)
Hispanic ethnicity	515 (17.9%)
Graft type	
Living donor	1,229 (42.9%)
Deceased donor	1,633 (57.1%)
Graft vintage, y	4.3 [1.7- 8.0]
Country	
United States	2,071 (71.7%)
Canada	378 (13.1%)
Brazil	440 (15.2%)
Hypertension	2,680 (92.8%)
Diabetes	1,145 (39.7%)
Prior myocardial infarction	407 (14.1%)
Prior stroke	198 (6.9%)
Pancreatic transplant	220 (7.6%)
eGFR by CKD-EPI, mL/min/1.73 m^2^	41 (11)
eGFR range, mL/min/1.73 m^2^	
45-<60	1,135 (39.3%)
30-<45	1,273 (44.1%)
<30	481 (16.6%)
UACR, mg/g	28 [10-119]
Immunosuppression regimen	
Prednisone, cyclosporine, MMF	797 (27.6%)
Prednisone, tacrolimus, MMF	667 (23.1%)
Prednisone, cyclosporine, azathioprine	295 (10.2%)
Prednisone, cyclosporine	234 (8.1%)
Prednisone, MMF	137 (4.7%)
Prednisone, tacrolimus	120 (4.2%)
Other (including prednisone)	395 (13.7%)
Other (prednisone-sparing)	244 (8.4%)

*Note:* N = 2,889. Values expressed as mean (standard deviation), number (percent), or median [interquartile range].

Abbreviations: CKD-EPI, Chronic Kidney Disease Epidemiology Collaboration; eGFR, estimated glomerular filtration rate; MMF, mycophenolate mofetil; UACR, urinary albumin-creatinine ratio.

Median follow-up time was 3.6 (IQR, 2.9-5.2) years. By 2 years of follow-up, 98 participants had experienced graft loss and 107 had died with a functioning graft. There were 129 participants (4.5% of the total study population) who were lost to follow-up or administratively censored before 2 years. By 5 years, 252 participants had experienced graft loss, 265 had died with a functioning graft, and 1,543 were lost to follow-up or administratively censored. Full distributions of KFRE-predicted risk by graft loss status are shown in Figure S2.

As shown in Table 2, the KFRE provided high discrimination at 2 years in the overall cohort (C statistic, 0.85; 95% CI, 0.81-0.88) and in subgroups of donor graft type and graft vintage, with C statistics ranging from 0.83 to 0.85. Discrimination for 5-year prediction was less (overall C statistic, 0.81; 95% CI, 0.78-0.84) but across subgroups still remained in a useful range from 0.78 to 0.82.

**Table 2:** C Statistics for 4-Variable KFRE Applied to Kidney Transplant Recipients in the FAVORIT Cohort

Population	n	2-y Outcomes		5-y Outcomes	
		Graft Loss Events	C Statistic (95% CI)	Graft Loss Events	C Statistic (95% CI)
Overall	2,889	98	0.85 (0.81-0.88)	252	0.81 (0.78-0.84)
Donor type					
Deceased	1,633	65	0.85 (0.81-0.89)	165	0.81 (0.78-0.84)
Living	1,229	30	0.83 (0.75-0.92)	82	0.80 (0.75-0.84)
Graft vintage					
<2 y	818	21	0.83 (0.77-0.90)	49	0.78 (0.72-0.84)
2-<5 y	768	21	0.83 (0.74-0.94)	64	0.80 (0.75-0.85)
≥5 y	1,285	54	0.85 (0.81-0.90)	135	0.82 (0.78-0.85)

*Note:* Subgroup counts do not sum to 2,889 due to missing data for donor type (n = 27) and graft vintage (n = 18).

Abbreviations: FAVORIT, Folic Acid for Vascular Outcome Reduction in Transplantation study; KFRE, Kidney Failure Risk Equation.

**Figure 2:**
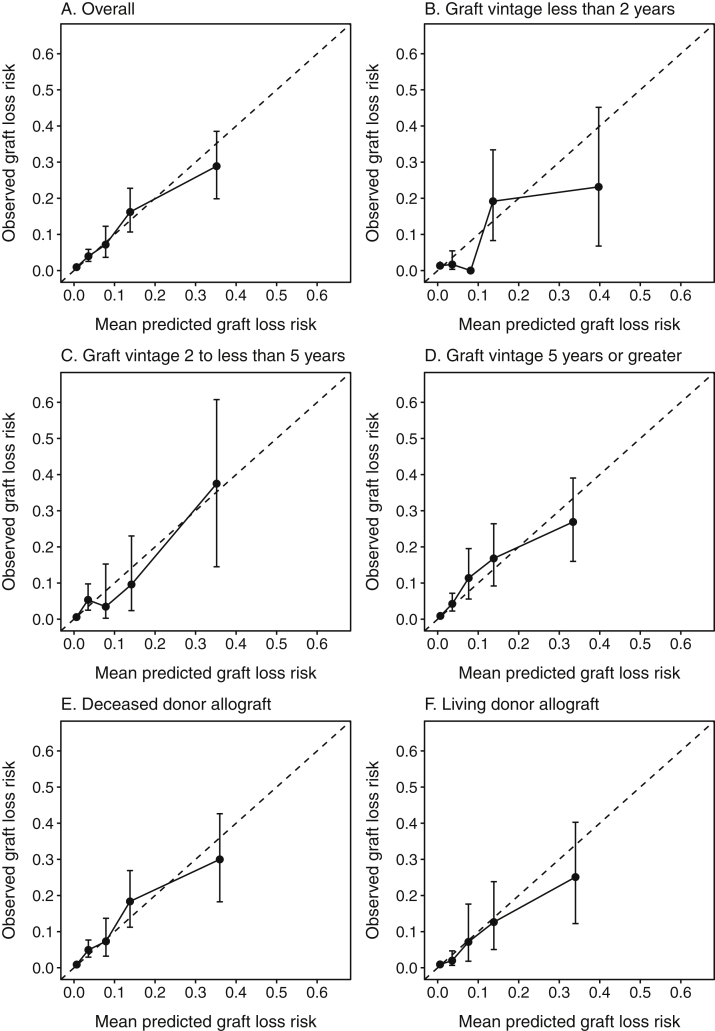
Observed versus predicted graft loss risk using 4-variable Kidney Failure Risk Equation at 2 years. The dotted line denotes perfect agreement between observed and predicted risk. Error bars represent 95% CIs for cumulative incidence of graft loss with return to dialysis within 2 years. (B) Error bars are not shown for the 6% to <10% predicted risk category because 0 of the 34 corresponding participants had experienced graft loss by the 2-year time point.

In the overall cohort, there was generally close agreement between predicted and observed risk for graft loss at 2 years (Fig 2) but with overestimation of risk in the highest risk category. Participants in the lowest predicted risk category (<2% predicted risk) had an observed risk for graft loss of 0.9% (95% CI, 0.6%-1.5%), whereas those in the highest predicted risk category had a mean predicted risk of 35.2% as compared with an observed risk for graft loss of 28.9% (95% CI, 19.9%-38.5%).

**Figure 3:**
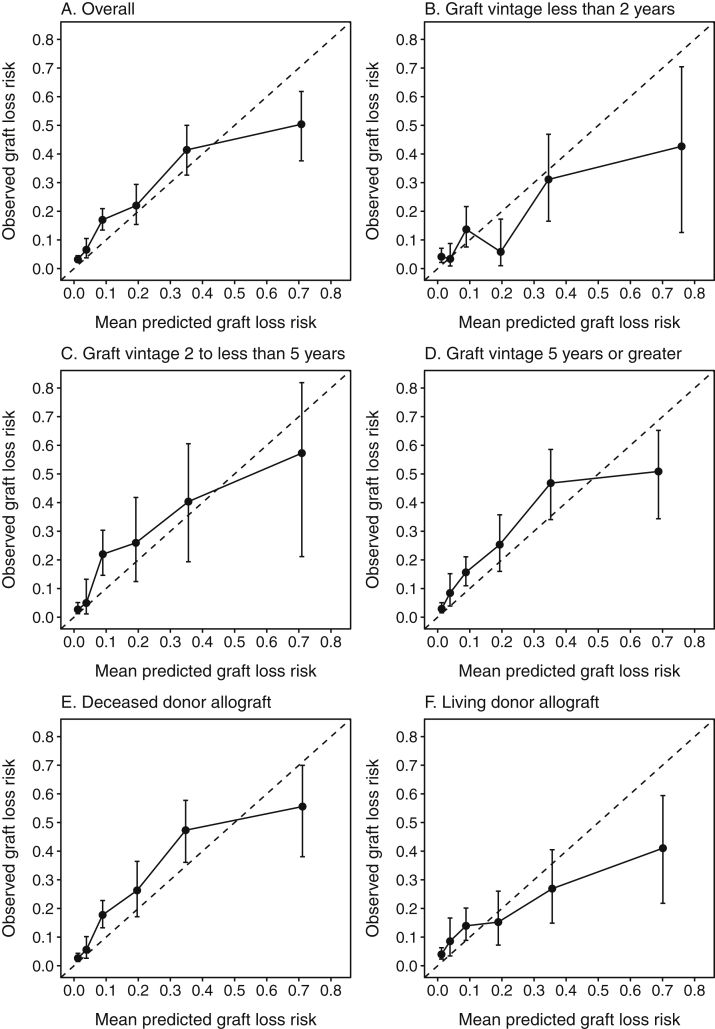
Observed versus predicted graft loss risk using 4-variable Kidney Failure Risk Equation at 5 years. The dotted line denotes perfect agreement between observed and predicted risk. Error bars represent 95% CIs for cumulative incidence of graft loss with return to dialysis within 5 years.

In subgroup analyses, the KFRE was well calibrated for both deceased donor and living donor grafts. Calibration was poorer for the subgroup with graft vintage less than 2 years, largely due to overestimation of predicted risk across multiple risk categories. The KFRE was relatively well calibrated in subgroups of graft vintage of 2 to less than 5 years and 5 years or greater. Calibration plots for 5-year predictions (Fig 3) showed overall adequate agreement between observed and predicted risk, but with a pattern of underestimation of risk in lower risk categories and overestimation of risk in the highest risk category. Overestimation of risk was particularly severe in subgroups of graft vintage less than 2 years and living donor grafts, for which mean predicted risk (75.9% and 70.1%, respectively) was nearly double the observed graft loss risk (42.7% and 41.0%, respectively).

In sensitivity analyses treating death as censoring, C statistics showed that minimally different discrimination and calibration was also similar, though overestimation of risk in the highest risk categories was slightly attenuated (Figs S3 and S4). In sensitivity analyses in which participants with eGFRs ≥ 60 mL/min/1.73 m^2^ were included, discrimination and calibration performances were not materially changed (Figs S5 and S6).

## Discussion

In this study, we found that the KFRE demonstrated accurate predictive performance in estimating the risk for graft loss at 2 years for kidney transplant recipients with eGFRs < 60 mL/min/1.73 m^2^, with the noted exception of poor calibration for participants less than 2 years posttransplantation. The KFRE has been evaluated for kidney transplantation in 2 prior studies, 1 examining 956 kidney transplant recipients from a single center in Canada (Akbari et al)^16^ and 1 examining 3,659 patients from 4 US and Canadian centers (Tangri et al).^34^ Both prior studies showed good to excellent discrimination with better discrimination for 2-year predictions compared with 5-year predictions, findings fairly comparable to our study. Akbari et al^16^ reported C statistics (assessed at varying time points posttransplantation) ranging from 0.73 to 0.93 for the 2-year KFRE and from 0.72 to 0.77 for the 5-year KFRE. In Tangri et al,^34^ C statistics were 0.81 and 0.73 for the 2- and 5-year KFREs, respectively, but in a subgroup of patients with eGFRs < 45 mL/min/1.73 m^2^, discrimination was higher (0.88 and 0.83, respectively). Notably, more than half (60.7%) of our study population had eGFRs < 45 mL/min/1.73 m^2^, and our C statistics of 0.85 and 0.81 at 2 and 5 years, respectively, are more comparable to this low eGFR subgroup.

Given that eGFR and albuminuria are independently powerful predictors of graft failure, the ability of the KFRE to predict accurately was unsurprising. However, our results highlight important limitations of the KFRE in the transplant setting. Although discrimination was in a clinically useful range overall for both 2- and 5-year predictions, the KFRE was not well calibrated for predictions in the setting of graft vintage less than 2 years. This is consistent with the findings of between-cohort variability in calibration in Tangri et al,^34^ in which prediction was assessed at the 1-year posttransplantation time point. In addition, we found that 5-year predictions tended to underestimate risk when risk is low and overestimate risk when risk is high, the latter being potentially severely discordant. Thus, unless risk is very low, the clinical usefulness of the KFRE is limited for counseling patients regarding long-term likelihood of graft loss, even when discrimination is acceptable.

Because numerous models have been developed for predicting graft loss in kidney transplant recipients, a key consideration for assessing the clinical applicability of prognostic models is the time of risk assessment, or the prognostic time origin, relative to the date of transplantation. This is the time point from which outcomes are defined (eg, 2-year risk or 5-year risk) and it is also the time point near which predictors (eg, eGFR and UACR) are typically ascertained. A systematic review of published risk prediction models for kidney allograft failure found that most models used predictors ascertained at or shortly after the time of transplantation (typically within 6-12 months posttransplantation).^15^

For instance, the Birmingham risk model, which uses recipient age, sex, ethnicity, eGFR, UACR, and prior acute rejection at 1 year posttransplantation, has demonstrated good to excellent discrimination (C statistics, 0.78-0.90 in validation cohorts) and good calibration for predicting 5-year risk for death-censored graft loss.^13^ This model has been subsequently refined with the addition of donor-specific alloantibody status and histologic data (presence of glomerulitis or chronic interstitial fibrosis), with further improvement in prediction.^14^

By contrast, few prediction models for graft loss have been validated that use any time point after transplantation as the prognostic time origin.^17^ Such models would be applicable for kidney transplant recipients who may be several years out from the date of transplantation and are often primarily monitored by general nephrologists in the community. In the setting of routine follow-up, similar to long-term monitoring of nontransplantation CKD, the ability to obtain updateable predictions for kidney failure within some time frame (eg, 2 years) after each visit would be well suited for advising patients and informing clinical decision making in real time. In the present study, the KFRE provided accurate predictions for kidney transplant recipients over a wide range of time points years after transplantation.

Given that most transplant centers refer kidney transplant recipients back to general nephrologists for comanagement within 12 months of transplantation, the KFRE may be advantageous in several ways.^35^ First, it is a simple prognostic model that general nephrologists may already be familiar with for assessing prognosis in nontransplantation patients with CKD. Second, the KFRE uses data routinely collected in nephrology care, and nephrologists in the community are likely to have up-to-date values readily available in local health records. Third, the simplicity of the KFRE makes it particularly amenable to automated reporting in electronic health records, and if implemented in this manner, it may alert clinicians to high-risk patients when there has not been a recent biopsy or DSA testing.

However, the KFRE should not replace more complex prognostic models that have been validated for kidney transplant recipients. For kidney transplant recipients who are within 1 year posttransplantation, it may be more appropriate to apply more detailed prognostic models that incorporate histologic and immunologic prognostic factors and that have demonstrated excellent performance in the early posttransplantation setting.^13^^,^^14^ For patients who are many years posttransplantation, the iBox model can be used if donor-specific antibody testing and biopsy data are available.^17^

Another limitation of the KFRE is that when eGFR is >60 mL/min/1.73 m^2^, predicted risk estimates are invariably low and thus add little to meaningful risk stratification necessary to inform clinical decisions. In this setting, prognostic models that incorporate additional transplant-specific predictors beyond age, sex, eGFR, and UACR and that were developed and validated without eGFR restriction may be more appropriate for risk stratification.^13^^,^^17^

Strengths of our study included the use of a relatively large multicenter validation cohort consisting of individuals with a wide range of graft vintages. This increased the applicability of our results because prediction of allograft failure did not require measurement of variables at a specific time posttransplantation. Also, there was little loss to follow-up for outcomes at 2 years. The rigorous use of analytical techniques to account for the competing risk for death when estimating C statistics and risks for graft loss was another strength. Because death with a functioning graft occurred with greater incidence than graft loss in our cohort, accounting for death as a competing event avoided bias due to censoring death, which would lead to overestimation of the absolute risk for dialysis.^15^^,^^36^

Our study had several limitations. First, no data were available for the reason for graft loss. The 5-year results should be interpreted with caution due to significant loss to follow-up. Another limitation was a relatively low crude rate of graft loss events within 2 years (98 of 2,889 [3.4%]). This may have been because FAVORIT enrolled participants with stable kidney function, many having been stable for several years posttransplantation. With such low rates of allograft loss within 2 years, high discrimination or calibration does not necessarily translate into clinically useful prognosis for patients beyond a reassurance that risk for graft failure is very low. However, this does not necessarily preclude the usefulness of risk prediction for other applications in which high discrimination is valuable, such as allocation of limited resources to those with highest risk, identification of high-risk individuals to optimize power in clinical trial enrollment, or surrogate end point development. FAVORIT eligibility criteria required participants to have elevated baseline serum homocysteine levels so it is unknown whether our results would apply to individuals without elevated homocysteine levels. In particular, homocysteine levels ≥ 12 μmol/L have been associated with increased risk for both graft loss and mortality in the kidney transplant population.^37^ This suggests that if the KFRE is accurate for kidney transplant recipients with elevated homocysteine levels, it may overestimate risk for those without elevated homocysteine levels. However, this selection criterion should not preclude generalizability of the result to most of the kidney transplant recipient population, which has been shown to have elevated homocysteine levels compared with the general population.^38^^,^^39^ Consistent with this, most (68%) of those who underwent eligibility screening for FAVORIT met the homocysteine level criterion for study entry.^21^

In conclusion, the KFRE provided accurate prediction for the risk for graft loss among prevalent adult kidney transplant recipients with eGFRs < 60 mL/min/1.73 m^2^ who are at least 2 years posttransplantation in a multicenter multinational setting. The KFRE is a simple and parsimonious tool for nephrologists to assess prognosis and aid decision making in routine follow-up of kidney transplant recipients many years after transplantation. Further studies are needed to assess the utility of the KFRE and whether its routine application to guide care for kidney transplant recipients approaching the end of their allograft life yields meaningful clinical benefits.

## Supplementary Material

Supplementary File (PDF) Figures S1-S6; Items S1-S2.
